# Comparative Evaluation of 24 LDL-C Estimation Equations Against Direct Assays in Two Independent Cohorts

**DOI:** 10.3390/diagnostics15182298

**Published:** 2025-09-10

**Authors:** Imola Györfi, Oana Roxana Oprea, Ion Bogdan Mănescu, Antoanela Curici, Minodora Dobreanu

**Affiliations:** 1 Doctoral School (I.O.S.U.D.), George Emil Palade University of Medicine, Pharmacy, Science, and Technology of Targu Mures, 540142 Targu Mures, Romania; imolag@yahoo.com; 2 Department of Laboratory Medicine, George Emil Palade University of Medicine, Pharmacy, Science, and Technology of Targu Mures, 540142 Targu Mures, Romania; bogdan.manescu@umfst.ro (I.B.M.); minodora.dobreanu@umfst.ro (M.D.); 3 Clinical Laboratory, Emergency County Clinical Hospital of Targu Mures, 540136 Targu Mures, Romania; 4 Department of Cellular and Molecular Biology and Histology, Carol Davila University of Medicine and Pharmacy, 020021 Bucharest, Romania; antoanela.curici@umfcd.ro; 5 Synevo Romania, 013681 Bucharest, Romania

**Keywords:** cardiovascular risk, equation, Friedewald, LDL-cholesterol, lipid profile

## Abstract

**Background:** Low-density lipoprotein cholesterol (LDL-C) is essential in diagnosing and managing dyslipidemias. While direct assays are faster than the reference beta-quantification method, many labs continue using the Friedewald (FW) equation, despite its limitations. **Methods:** Two large datasets were analyzed: 10,174 hospital samples (Cobas/Roche) and 21,091 private lab samples (Alinity/Abbott). Various literature-based LDL-C equations were compared, focusing on FW, Sampson (SN), and Martin–Hopkins (MH). Direct LDL-C served as the reference. Evaluation metrics included bias and classification accuracy. **Results:** In samples with triglycerides < 400 mg/dL, several lesser-known equations showed acceptable bias (±5%), outperforming FW, SN, and MH, which had biases from −7.4% to −4.9%. Classification accuracy was higher with equations like Vujovic (up to 82.5%), compared to FW (65.8%), SN (73.1%), and MH (72.4%). The Vujovic equation showed minimal bias across triglyceride levels and the highest net gain in correct classification (3.4% and 1.57%). **Conclusions:** Multiple lesser-known LDL-C formulas outperformed widely used ones. The Vujovic equation yielded the best results, but the limited net clinical improvement suggests that replacing Friedewald may not be urgently necessary.

## 1. Introduction

LDL cholesterol (LDL-C) can be measured using a variety of methods. Beta-quantification is the reference method for measuring LDL-C. However, this technique is expensive, laborious and time-consuming, making it impractical for routine use in clinical laboratories, where speed and cost-effectiveness are essential considerations. Consequently, the LDL-C concentration can be determined in two ways: direct clearance/enzymatic method or mathematical estimation. Although the enzymatic method comes with additional costs, it provides faster results than ultracentrifugation and offers greater accuracy compared to estimation equations. However, direct measurement has its own limitations, and studies suggest that significant differences may exist in patients with dyslipidemia and patients undergoing statin treatment [1,2,3,4,5,6].

Many clinical laboratories choose to calculate LDL-C concentration using the Friedewald equation, first published in 1972, in order to reduce costs [7]. However, this method has limitations: it cannot be applied when triglyceride levels exceed 400 mg/dL [7,8] and tends to be less accurate the higher the triglyceride concentration and at very low LDL-C levels [9,10]. Consequently, the Friedewald equation requires fasting, which may represent a disadvantage in routine clinical practice. Additionally, it relies on a “one-size-fits-all” assumption, using a fixed factor (TG/5) to estimate VLDL cholesterol, which fails to account for inter-individual variation and is markedly inaccurate in conditions such as dysbetalipoproteinemia. Moreover, the Friedewald equation does not account for cholesterol contained in lipoprotein remnants, such as intermediate-density lipoproteins and chylomicron remnants.

Because of these limitations, researchers have sought to develop more accurate equations for LDL-C estimation. Numerous such formulas have been validated globally, with varying degrees of accuracy [11,12,13]. Among them, the Sampson [14] and Martin–Hopkins [15] equations have gained prominence and have begun to be implemented in clinical laboratories worldwide.

The Sampson equation, a more recent and mathematically complex alternative to the Friedewald method, aims to improve LDL-C estimation by incorporating additional adjustments for variations in lipid profiles [14]. However, like Friedewald, it still follows a “one-size-fits-all” approach, using a fixed equation. Thus, while the Sampson equation provides improved accuracy, particularly for patients with dyslipidemia or very high triglyceride levels, it still does not account for the individual variability in lipid metabolism.

The Martin–Hopkins equation improves accuracy by using a patient-specific adjustment based on triglyceride and non-HDL-cholesterol levels [15]. It employs a detailed table with over 180 cells to better estimate VLDL cholesterol, offering a more personalized approach than the fixed ratio used in Friedewald’s method. This equation is particularly useful for patients with higher triglyceride levels or low LDL-C values, as it provides a refined calculation that reflects individual lipid profiles more accurately. In its original form, the Martin–Hopkins equation, like the Friedewald formula, is applicable only for triglyceride concentrations up to 400 mg/dL. The extended Martin–Hopkins equation, similar to the Sampson equation, permits LDL-C estimation at triglyceride levels up to 800 mg/dL [16]. Nevertheless, even these specifically designed equations demonstrate reduced accuracy and higher rates of misclassification when triglycerides exceed 400 mg/dL [17].

We are currently living in an era of highly effective lipid-lowering therapies, with clinical guidelines recommending LDL-C targets as low as below 55–70 mg/dL for individuals at high or very high cardiovascular risk [18,19,20]. LDL-C values play a central role in cardiovascular risk stratification, in setting therapeutic targets at the time of diagnosis, and in monitoring treatment efficacy and patient adherence over time. As such, accurate LDL-C estimation is important for both clinicians and patients. The clinical laboratory bears the primary responsibility for ensuring that LDL-C results are reliable and consistent. Consequently, the topic of LDL-C measurement and estimation methods has remained highly relevant over the years and continues to be a matter of scientific and clinical interest, especially among laboratory professionals.

In this study, we aimed to compare the accuracy of published LDL-C estimation equations with that of directly measured LDL-C values obtained using two widely used analytical platforms: Abbott Alinity and Roche Cobas.

## 2. Materials and Methods

This retrospective observational study was conducted on patient laboratory records obtained between January 2023 and February 2025. The dataset included results from 31,265 individuals, comprising inpatients and outpatients from a tertiary care hospital (Database 1, *n* = 10,174) and outpatients from a private clinical laboratory (Database 2, *n* = 21,091). The study was approved by the Ethics Committee of the tertiary care hospital from which part of the laboratory data was obtained (approval no. 19727/27.07.2023), and written permission was granted by the private laboratory that provided the remaining dataset.

Patients were included if their laboratory record contained a complete standard lipid profile performed from a single blood sampling event, including total cholesterol (TC), high-density lipoprotein cholesterol (HDL-C), triglycerides (TG), and directly measured low-density lipoprotein cholesterol (dLDL-C). Non-HDL-cholesterol (non-HDL-C) was calculated as a difference between TC and HDL-C. Remnant cholesterol (RC) was calculated as TC minus HDL-C and dLDL-C.

Direct LDL-C measurement was performed using a selective direct LDL assay on the Cobas pure platform (Roche Diagnostics, Basel, Switzerland) in the hospital laboratory, and a liquid selective detergent assay on the Alinity ci system (Abbott Laboratories, Abbott Park, IL, USA) in the private laboratory. Both platforms employed direct homogeneous enzymatic assays standardized against beta-quantification, which is considered the reference method for LDL-C determination. Both assays demonstrated excellent analytical performance, with Limit of Blank, Limit of Detection, and Limit of Quantification values of 3.87 mg/dL for Roche and 0 mg/dL for Abbott; analytical measurement range of 3.87–549 mg/dL (Roche) and 1–800 mg/dL (Abbott); and within-laboratory imprecision of <2.5% CV (Roche) and <1.6% CV (Abbott). The calculation of LDL-C was performed using 24 different estimation equations, as detailed in Supplementary Table S1 [7,14,15,16,21,22,23,24,25,26,27,28,29,30,31,32,33,34,35,36,37,38,39,40,41].

Directly measured LDL-C was used as the reference in the analysis. To assess the accuracy of the estimated values, the percentage difference (%Delta) was calculated with the formula: %Delta = 100 × (Calculated LDL-C − dLDL-C)/dLDL-C. The %Delta was used to assess how much each calculated LDL-C value differed from the dLDL-C, providing a practical measure of bias for each equation. For interpretative purposes, performance thresholds were defined as follows: good (%Delta between −5% and +5%), moderate (%Delta −10% to −5% or +5% to +10%), low (%Delta −20% to −10% or +10% to +20%), and unacceptable (%Delta less than −20% or greater than +20%). These categories were established arbitrarily to facilitate comparative evaluation of equation accuracy. Statistical analyses were conducted using MedCalc^®^ Statistical Software version 20.104 (MedCalc Software Ltd., Ostend, Belgium).

The accuracy of the LDL-C estimation equations was further evaluated in terms of categorical agreement. Based on established clinical decision thresholds and guideline-recommended LDL-C targets, patients were stratified into eight categories: <40, 40–54, 55–69, 70–99, 100–129, 130–159, 160–189, and ≥190 mg/dL. For each equation, the calculated LDL-C value was compared to the directly measured LDL-C (dLDL-C) to determine whether both values placed the patient in the same clinical category. The agreement rate (defined as the proportion of cases where the calculated LDL-C matched the dLDL-C category) was computed for each individual interval, as well as overall, across all 24 equations.

## 3. Results

### 3.1. Demographic and Laboratory Characteristics

Demographic and laboratory parameters were compared between the two datasets, and the results are presented in Table 1. The hospital clinical laboratory cohort included a slightly higher proportion of male patients and an older population overall. Despite this, significantly lower median concentrations were observed for TC, dLDL-C, non-HDL-C in the hospital group. No significant differences were found between the two groups in terms of TG, HDL-C, or RC. Additionally, the proportion of patients with TG ≥ 400 mg/dL was nearly identical between the two laboratories.

### 3.2. Calculated and Measured LDL-C—Accuracy of LDL-C Equations

**Figure 1:**
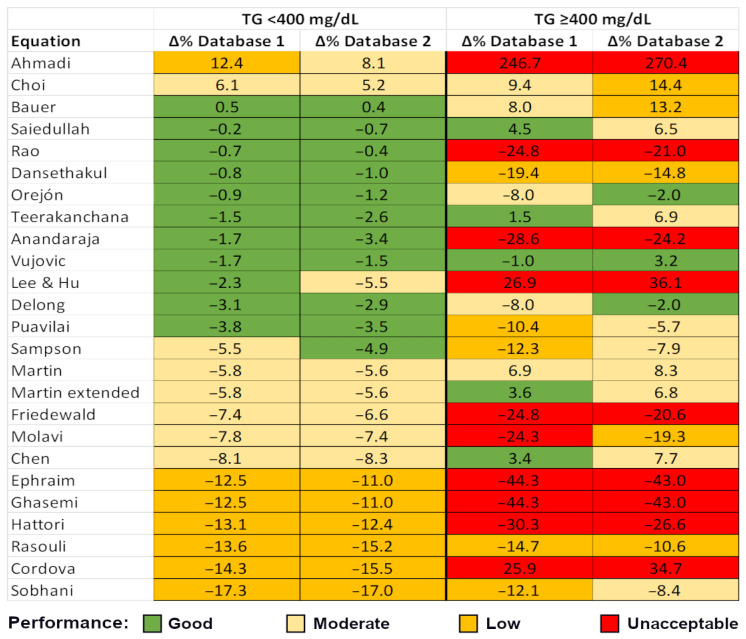
A clear, color-coded hierarchical representation of the equations’ performance. The list is ordered by the TG < 400 mg/dL category (representing the majority of data), first by Database 1, and then by value, from the highest (positive) to the lowest (negative) median bias. Equation performance is color-coded according to median error (%): within ±5%—good performance (green); within 5–10%—moderate performance (light orange); within 10–20%—low performance (orange); and greater than ±20%—unacceptable (red). In the TG < 400 mg/dL group, the equation ranking is largely preserved; however, in the smaller TG ≥ 400 mg/dL group, the ranking becomes inconsistent. The Vujovic equation [41] stands out as the only formula demonstrating good performance across all TG values and in both databases. This figure should be interpreted alongside Supplementary Tables S2 and S3. Equation references: Ahmadi [21], Choi [25], Bauer [23], Saiedullah [38], Rao [36], Dansethakul [27], Orejón [34], Teerakanchana [40], Anandaraja [22], Vujovic [41], Lee&Hu [32], Delong [28], Puavilai [35], Sampson [14], Martin [15], Martin extended [16], Friedewald [7], Molavi [33], Chen [24], Ephraim [29], Ghasemi [30], Hattori [31], Rasouli [37], Cordova [26], Sobhani [39].

The accuracy of the 24 LDL-C estimation equations was assessed using the percentage difference (%Delta), as defined in the Methods section. Given the known impact of hypertriglyceridemia (TG ≥ 400 mg/dL) on LDL-C calculation, the analysis was stratified into two groups: samples with TG < 400 mg/dL (Supplementary Table S2, Figure 1) and samples with TG ≥ 400 mg/dL (Supplementary Table S3, Figure 1). The vast majority of samples fell into the TG < 400 mg/dL group. A clear, color-coded hierarchical representation of the equations’ performance is presented in Figure 1.

In samples with triglyceride levels ≥ 400 mg/dL, many LDL-C estimation equations exhibited a positive median bias relative to dLDL-C, with median %Delta values reaching up to +270% (Ahmadi [21], Figure 1). The three most commonly used equations (Friedewald [7], Martin–Hopkins [15], and Sampson [14]) demonstrated reduced accuracy under hypertriglyceridemic conditions compared to their performance in samples with TG < 400 mg/dL. Nevertheless, the extended Martin–Hopkins equation showed improved accuracy in hypertriglyceridemic patients, with good and moderate performances, respectively (Figure 1).

### 3.3. Calculated and Measured LDL-C—Categorical Agreement for Risk Stratification

For risk stratification purposes, a categorical agreement analysis was conducted between calculated LDL-C values and dLDL-C, using eight clinically relevant LDL-C categories ranging from <40 mg/dL to ≥190 mg/dL. The results for each database are presented in Table 2 (Database 1) and Table 3 (Database 2). It is important to note that this agreement analysis was performed across all samples, irrespective of triglyceride concentration.

In line with their previously discussed accuracy, several equations demonstrated strong categorical agreement with the dLDL-C method, although correct classification rates were consistently lower in Database 2. Among the best-performing equations were: Bauer (82.5% and 77.3%), Vujovic (81.9% and 76.6%), Orejón (81.7% and 75.6%), Teerakanchana (80.4% and 71.3%), Saiedullah (79.0% and 75.5%), Rao (78.4% and 73.0%), Dansethakul (79.4% and 74.1%), and Delong (78.9% and 74.5%). The widely used Friedewald equation showed a more modest categorical agreement, with correct classification rates of 65.8% and 64.5%. Martin–Hopkins (72.4% and 69.1%) and Sampson (73.1% and 70.2%) performed better than Friedewald but still fell short of the top-performing equations. There was no meaningful difference in LDL-C classification accuracy between the Martin–Hopkins equation and its extended version, with only a 0.1% difference observed in both databases.

All equations that demonstrated superior overall LDL-C classification accuracy compared to the Friedewald equation (as shown in Table 2 and Table 3) were included in a subsequent analysis to quantify the extent of their improvement in correctly classifying individuals. The detailed results of this analysis are presented in Supplementary Tables S4 and S5.

**Figure 2:**
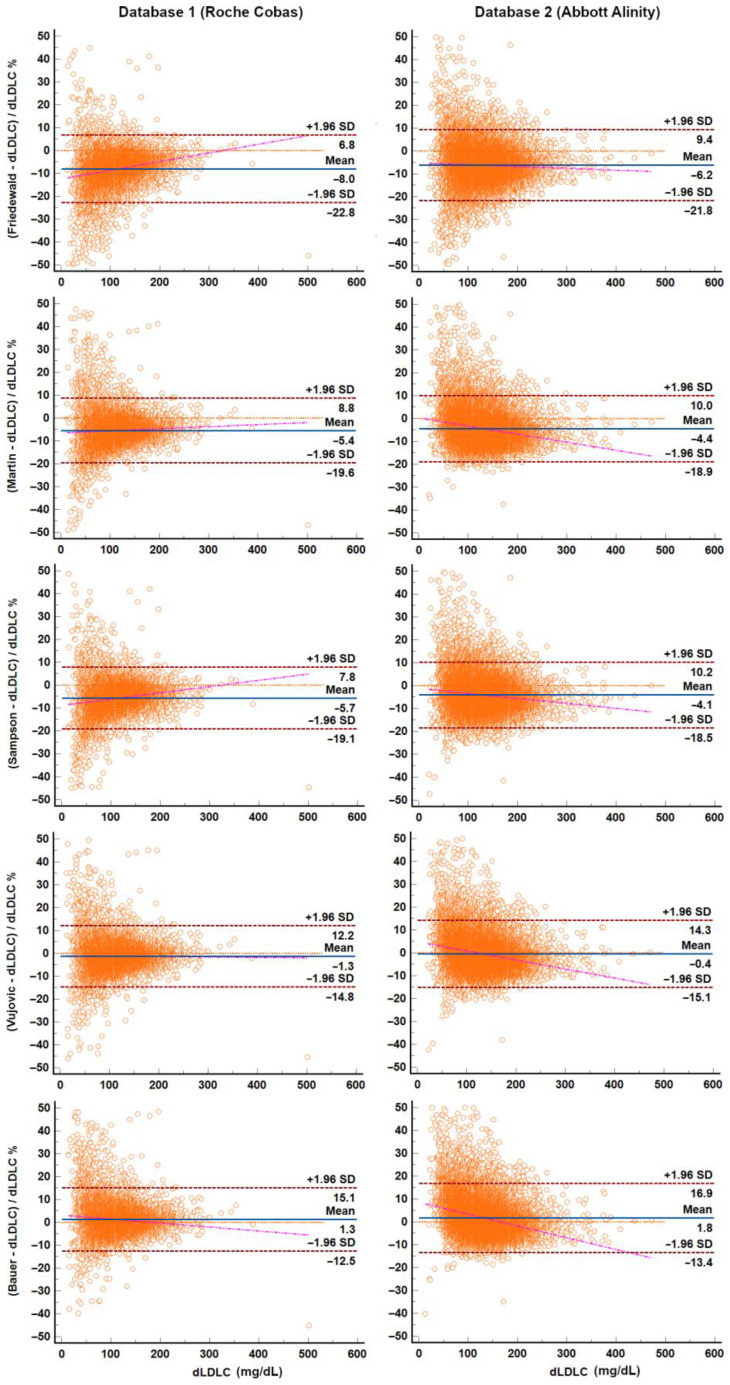
Bland–Altman plots for the most widely used equations (Friedewald, Martin, and Sampson) and the top-performing equations (Vujovic: lowest error; Bauer: highest classification accuracy). For practical reasons related to *Y*-axis scaling, datapoints with a % bias relative to dLDL-C ≥ 50% were excluded, while still retaining the vast majority of datapoints. Only lipid profiles with TG levels < 400 mg/dL were included in the analysis.

**Figure 3:**
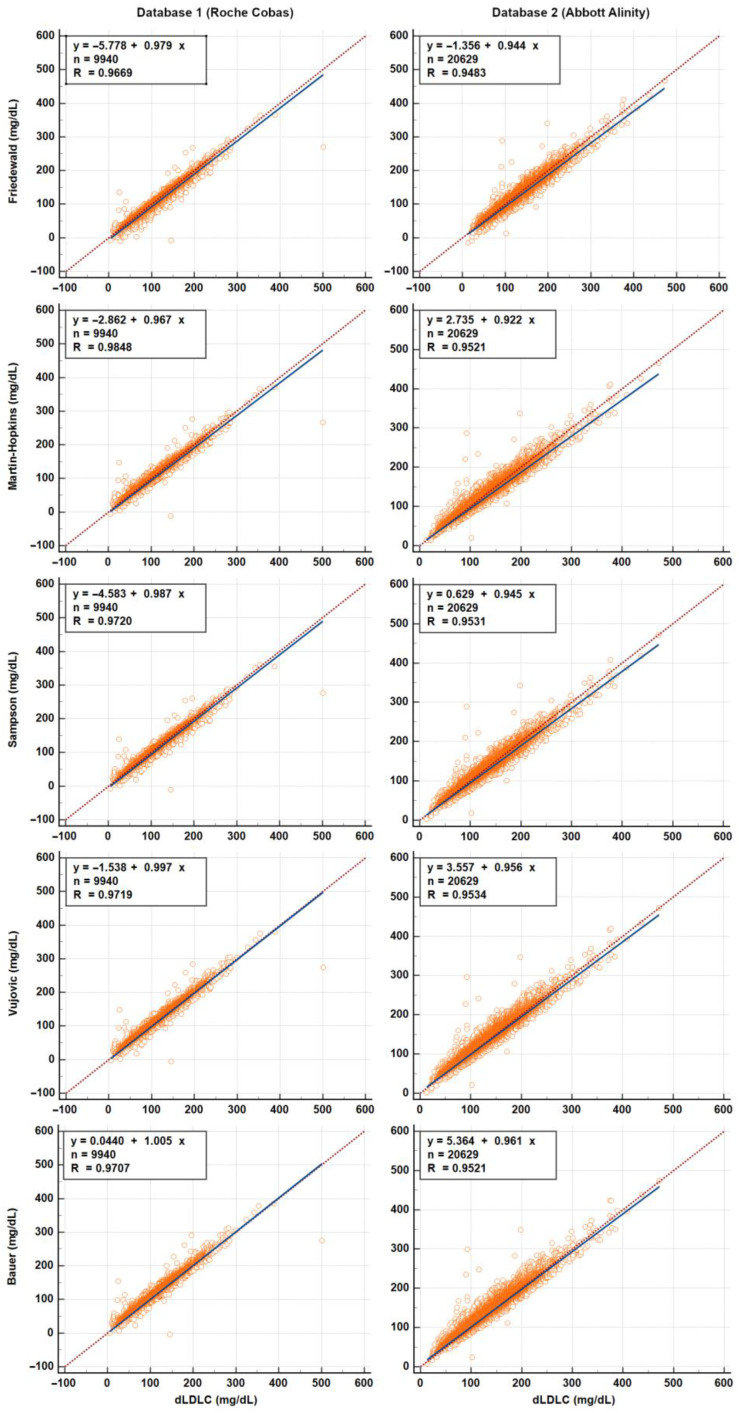
Passing–Bablok plots for the most widely used equations (Friedewald, Martin, and Sampson) and the top-performing equations (Vujovic: lowest error; Bauer: highest classification accuracy). Only lipid profiles with TG levels < 400 mg/dL were included in the analysis.

For clarity, Bland–Altman and Passing–Bablok plots are presented in Figure 2 and Figure 3, respectively, for the most commonly used equations (Friedewald, Martin–Hopkins, and Sampson), as well as for the Vujovic and Bauer equations, which showed the best performance in bias and classification analyses, respectively.

## 4. Discussion

### 4.1. Advantages and Limitations of Direct Assays

The methods employed in our study for LDL-C determination in both laboratories were direct homogeneous assays: the Roche method for Database 1 and the Abbott method for Database 2. Although direct assays are convenient and widely adopted in clinical laboratory practice, they are not regarded as the reference standard. The gold-standard method for LDL-C quantification remains beta-quantification via ultracentrifugation.

Martins et al. have noted that a principal limitation of dLDL-C assays is the absence of methodological standardization across laboratories, with performance being highly dependent on the specific assay and reagents utilized [5]. Similarly, Steyn et al. raise concerns regarding the reliability of direct methods, questioning whether their limitations justify their routine use over calculated LDL-C using validated predictive equations [3]. Both studies further emphasize the significant additional costs associated with direct assays [3,5].

Numerous reports indicate that the accuracy of dLDL-C assays may be compromised under certain clinical conditions, such as very low LDL-C concentrations, elevated lipoprotein(a), or ongoing lipid-lowering therapies (e.g., statins or PCSK9 inhibitors) [1,2,3,4,5,6]. Several studies have documented discrepancies in such scenarios. For instance, Miida et al. demonstrated that certain homogeneous assays yielded less accurate results in dyslipidemic patients compared to beta-quantification [42]. An independent evaluation of the Kyowa Medex (Sekisui) selective detergent method also revealed diminished accuracy in hyperlipidemic samples [43]. Conversely, a brief report by Rodríguez-Domínguez et al. concluded that the Beckman Coulter dLDL-C assay performs reliably in samples with triglyceride concentrations ≥ 400 mg/dL or LDL-C < 70 mg/dL [8]. Cemin et al. observed that the Roche dLDL-C method tended to overestimate LDL-C levels in high cardiovascular risk patients [6]. However, this overestimation was relative to values derived from the Friedewald and Sampson equations, both of which are recognized for underestimating LDL-C [6]. Thus, we posit that the apparent overestimation reported by Cemin et al. may not signify a true limitation of the Roche method, but rather a potential advantage, as it may offer a closer approximation to the actual LDL-C concentration.

Overall, while direct assays are generally considered more accurate than calculated LDL-C, the literature presents mixed evidence, with assay performance varying based on patient characteristics, clinical context, and assay type. Some dLDL-C methods may yield reliable results in specific populations yet fall short in others. Consequently, investigating the limitations of direct methods is essential and should be undertaken at the institutional level. Each laboratory should conduct internal validation studies to identify method-specific limitations, evaluate cost-effectiveness, and determine the appropriateness of routine implementation.

### 4.2. Direct Assays as De Facto Reference Methods

While beta-quantification remains the gold standard for LDL-C measurement, it is not practical for routine use in clinical laboratories as it is laborious, costly, and time-consuming. However, most dLDL-C methods are standardized and traceable to beta-quantification, which makes them a reliable alternative for routine use in clinical laboratories [1,20,21,22]. In everyday clinical practice, when there is uncertainty about the accuracy of calculated LDL-C, clinicians often request dLDL-C testing. Consequently, dLDL-C measurement is frequently regarded as the most accurate method available in routine clinical practice, serving as a de facto reference among commonly used techniques due to its rapid turnaround time and accessibility for clinicians. Therefore, comparing calculated LDL-C values from different equations with dLDL-C may be justified, as this approach reflects what is routinely done in clinical care.

Using dLDL-C assays as reference methods is not uncommon in clinical and research settings, despite their distinction from the gold-standard beta-quantification method. For instance, among the 24 LDL-C estimation equations evaluated in this study, at least 14 were derived using statistical analyses in which dLDL-C served as the reference method. These include equations developed by Bauer [23], Chen [24], Choi [25], and Dansethakul [27] (based on Roche assay); Cordova [26] and Lee & Hu [32] (Wako assay); Ghasemi [30] and Rasouli [37] (Pars Azmon Inc. assay); Anandaraja [22] (Beckman assay); Ahmadi [21] (Technicon assay); Orejón [34] (Siemens assay), and Vujovic [41] (Kyowa Medex assay). Additionally, the equations proposed by Ephraim [29] and Teerakanchana [40] were also constructed using dLDL-C measurements as reference values, but the specific assay employed was not reported.

### 4.3. Differences Between Laboratory Databases

According to Table 1, although the patients from the hospital laboratory (Database 1) were generally older, they had lower levels of TC, LDL-C, and non-HDL-C compared to those from the independent laboratory (Database 2). This could be explained by the fact that many hospital patients had diagnosed dyslipidemia and cardiovascular disease and were already receiving treatment and regular follow-up. In contrast, individuals tested in the independent laboratory may have included more asymptomatic or undiagnosed cases, given the nature of this private laboratory.

### 4.4. Performance and Bias of LDL-C Equations

In our analysis, the performance of 24 LDL-C estimation equations, gathered from the literature, was compared with LDL-C values obtained using the direct enzymatic method on two platforms (Alinity and Cobas), which were used as the reference. The performance was assessed based on the median %Delta between estimated and directly measured values as a key indicator of bias.

As shown in Figure 1, in the larger subgroup analyzed (TG < 400 mg/dL), both laboratories demonstrated a similar performance ranking of the LDL-C equations. Specifically, the equations by Ahmadi, Choi, and Bauer showed a consistent positive median bias compared with dLDL-C, while the remaining equations exhibited varying degrees of negative median bias, as low as −17% in the case of Sobhani’s equation.

Among the most widely used equations, a moderate negative median bias was observed in both datasets: Friedewald (−7.4% and −6.6%), Martin–Hopkins (−5.8% and −5.6%), and Sampson (−5.5% and −4.9%). Notably, several equations achieved a median bias of less than 5%, which was considered good performance. Specifically, the equations proposed by Bauer, Saiedullah, Rao, Dansethakul, Orejón, Teerakanchana, Anandaraja, Vujovic, Delong, and Puavilai demonstrated good performance, with median bias values falling within the ±5% range. Some equations, such as those of Sampson and Lee & Hu, exhibited good performance in one dataset and moderate performance (bias between 5% and 10%) in the other. Several equations showed intermediate accuracy, with median bias values consistently between 5% and 10% (including the widely used Friedewald equation). In contrast, the equations developed by Ephraim, Ghasemi, Hattori, Rasouli, Cordova, and Sobhani were the least accurate, displaying low performance characterized by a marked negative bias (median %Delta below –10%) in both datasets, and ranked in that same descending order of accuracy. Importantly, among samples with triglyceride levels below 400 mg/dL, none of the equations exhibited a median bias exceeding ±20%. Therefore, according to the classification criteria defined in the Methods section, no equation was categorized as unacceptable within this TG range.

However, the performance landscape changed notably in the smaller subgroup with triglyceride levels ≥ 400 mg/dL. The Friedewald equation exhibited a marked increase in negative bias, with median %Delta values of −24.8% and −20.6%, both falling within the unacceptable range. In contrast, the Sampson equation demonstrated a more modest reduction in accuracy, with median %Delta values of −12.3% (low performance) and −7.9% (moderate performance), indicating that it may still offer clinically acceptable estimations in this context. These findings align with existing knowledge: the Friedewald equation was not developed for use in hypertriglyceridemic conditions and is not recommended for TG levels above 400 mg/dL [7], whereas the Sampson equation was specifically designed to be more robust under such conditions [14].

In contrast to the increasingly negative bias observed with the Friedewald and Sampson equations, the Martin–Hopkins equation displayed a shift from negative to positive median bias in the high-TG subgroup, with %Delta values of +6.9% and +8.3%, both within the moderate performance range, suggesting reasonable accuracy. This phenomenon has been previously observed in our earlier work on a smaller cohort, where the Martin–Hopkins equation exhibited the greatest tendency for bias to shift toward positive values with increasing TG concentrations, even within the TG <400 mg/dL range [44]. In contrast, the Friedewald and Sampson equations demonstrated more stable performance and were less influenced by rising triglyceride levels within this range [44]. The same phenomenon was noted by Drobnik et al. in a more recent study involving 5738 databases, which observed a progressively stronger positive bias in the Martin–Hopkins equation with rising triglyceride concentrations [45]. The extended Martin–Hopkins equation also exhibited a positive bias, although the magnitude was reduced compared with the original Martin equation: 3.6% (good performance) vs. 6.9% (moderate performance) in Database 1, and 6.8% vs. 8.3% (both moderate performance) in Database 2. This supports its intended purpose, as the extended Martin–Hopkins equation was specifically designed for samples with elevated triglyceride concentrations of up to 800 mg/dL [16].

Furthermore, consistent with observations in the TG < 400 mg/dL subgroup, several equations demonstrated acceptable performance even in hypertriglyceridemic samples (TG ≥ 400 mg/dL), achieving good performance in one dataset and moderate in the other. These included the equations proposed by Saiedullah, Orejón, Teerakanchana, Delong, and Chen. Notably, the Vujovic equation stood out for its consistent accuracy, maintaining good performance (defined as a median bias within ±5%) across all triglyceride ranges and in both datasets. Also, the Vujovic equation is both user- and laboratory-friendly due to its simple, Friedewald-like format. The only distinction lies in the treatment of triglycerides, which are divided by 6.85 instead of 5.00. Upon closer examination, a common trait emerges among the majority of these top-performing equations: the coefficients applied to TC, HDLC, and particularly TG are generally lower than those used in the traditional Friedewald formula, that is below 1.00 for TC and HDLC, and below 0.20 for TG (Supplementary Table S1). This approach resembles, to some extent, the Martin–Hopkins equation, in which the TG multiplier is individualized and decreases progressively as TG concentrations increase [15]. While such adjustments might appear tailored specifically to mitigate the underestimation of LDL-C in high-TG samples, it is important to note that, except for Chen, these equations did not perform well exclusively in the high-TG subgroup. In fact, they were also among the top-performing equations in the TG < 400 mg/dL cohort, suggesting a more generalized robustness in their predictive accuracy.

It is worth noting that although the Martin–Hopkins and Sampson equations are frequently cited in the literature as among the most accurate across diverse populations, particularly in complex scenarios such as low LDL-C or elevated triglyceride levels, the present study found these equations to be moderate performers rather than top-tier. This discrepancy may be attributable to several factors, including population-specific characteristics and, perhaps more importantly, methodological differences. For example, a large-scale study conducted by Samuel and colleagues, which analyzed over five million samples, identified the Martin–Hopkins and Sampson equations as the most accurate [11]. However, their evaluation relied on categorical classification according to LDL-C guideline thresholds, rather than on a continuous bias metric such as %Delta. Nevertheless, many of the top-performing equations in our analysis also demonstrated strong performance in the study by Samuel and collaborators [11]. A more detailed discussion will follow in the subsequent section.

Lastly, regardless of their median %Delta performance, all LDL-C estimation equations exhibited instances of critical underestimation or overestimation, independent of triglyceride concentration. In samples with TG < 400 mg/dL, %Delta values ranged from −375% (Ghasemi/Ephraim) to +1167% (Ahmadi), as presented in Table 2. The discrepancies were even more pronounced in the TG ≥ 400 mg/dL subgroup (Table 3), with %Delta values extending from −1440% (Ghasemi/Ephraim) to +8692% (Ahmadi). Importantly, even the most commonly used equations showed substantial individual errors. For instance, within the TG < 400 mg/dL group, the Friedewald equation produced %Delta values between −270% and +446%, Martin–Hopkins ranged from −254% to +496%, and Sampson from −267% to +464%. These findings underscore the potential risk of clinically significant misclassification when relying solely on calculated LDL-C values. They also demonstrate the limitation of using only median %Delta as a metric for clinical validation of such equations. In a previous smaller cohort, we hypothesized that such extreme outliers could result from a combination of factors affecting equation accuracy; however, this hypothesis warrants further investigation in a larger cohort [44].

### 4.5. Classification Performance of LDL-C Equations

While continuous bias analysis offers valuable insight into equation performance, the clinically most relevant metric for evaluating an LDL-C estimation method is its ability to correctly classify patients into appropriate risk categories, as compared with the reference method (in this case, dLDL-C). To address this, we conducted a categorical analysis using the nine LDL-C intervals defined in the Methods section. The classification accuracy results are presented in Table 2 (Database 1) and Table 3 (Database 2) and were calculated across all samples, regardless of TG levels. It is worth noting, however, that the proportion of patients with TG ≥ 400 mg/dL was relatively low in both datasets (approximately 2%).

This analysis revealed modest classification accuracies for the most widely adopted equations: Friedewald (65.8% and 64.5%), Martin–Hopkins (72.4% and 69.1%), extended Martin–Hopkins (72.5% and 69.0%), and Sampson (73.1% and 70.2%). By contrast, several lesser-known equations, many of which had also demonstrated superior performance in the bias analysis, achieved substantially higher classification accuracies, often in the 75–82% range. Notable examples include Bauer, Vujovic, Orejón, Saiedullah, Dansethakul, and Delong. These findings diverge from those reported by Samuel and colleagues, who observed much higher classification accuracies for these same three equations in a large-scale study involving over five million samples: 83.2% for Friedewald, 86.3% for Sampson, and 89.6% for Martin–Hopkins. The key difference likely lies in the reference method used: whereas the present study employed direct homogeneous assays, Samuel et al. used vertical auto profile ultracentrifugation, a more accurate technique [11].

Despite methodological differences, our results do converge with Samuel’s study in certain areas. Several top-performing equations overlapped across both analyses. For instance, Puavilai achieved classification accuracies of 84.1% in Samuel’s work versus 77.4% and 73.1% in our work; Delong reached 83.3% there versus 78.9% and 74.5% here; Saiedullah attained 82.1% compared to 79.0% and 75.5%; and Vujovic remained consistently strong with 80.3% in their study and 81.9% and 76.6% in ours. Notably, the Bauer equation, ranked only 13th in Samuel’s study with an accuracy of 73.8%, emerged as the top performer in our analysis, with classification accuracies of 82.5% and 77.3% in the two datasets, respectively.

Interestingly, although Friedewald, Martin–Hopkins, and Sampson demonstrated lower overall classification accuracy, they performed particularly well in the lowest LDL-C category (<40 mg/dL) within Database 1, achieving accuracies of 88.7%, 81.7%, and 87.4%, respectively. These accuracies closely mirror the overall performance of these equations as reported by Samuel et al. and are notably higher than the accuracies they observed specifically in the low LDL-C category (<40 mg/dL), which were 42.1%, 78.6%, and 66.0%, respectively [11]. However, this strong performance was not replicated in Database 2, where accuracies dropped markedly to 57.1%, 61.9%, and 67.9%. These discrepancies underscore the substantial impact that methodological variation, particularly the choice of dLDL-C assay, can have on the evaluation of equation performance, especially at clinically relevant LDL-C thresholds.

### 4.6. Net Gain in Classification Accuracy: Is It Enough to Replace the Friedewald Equation?

Only equations that outperformed the Friedewald formula in overall LDL-C classification accuracy were selected for further analysis to quantify their improvement in classification. This analysis, summarized in Supplementary Tables S4 and S5, was conducted using the 70 mg/dL threshold, both due to its clinical relevance as defined by current guidelines [18] and because of the well-documented decline in Friedewald’s accuracy at lower LDL-C concentrations (commonly defined as <70 mg/dL) [15]. In Database 1, all twelve equations demonstrated high concordance with Friedewald, with classification agreement rates ranging from approximately 90% to 98%. However, in a small subset of cases (0.3% to 4.0%), Friedewald correctly classified LDL-C while the comparator equation did not. Importantly, none of the superior equations resulted in downward reclassification below the 70 mg/dL threshold; rather, all led to upward reclassification in 1.8% to 5.9% of cases. The net gain in correct classification ranged from 1.5% for the Sampson equation to 3.6% for the Bauer equation. Although numerically modest, such improvements may be clinically meaningful and could support the adoption of alternative equations over Friedewald in routine practice.

In Database 2, the concordance between Friedewald and the alternative equations was even higher than in Database 1, ranging from 94% to 98%. This led to fewer instances where Friedewald provided superior classification (0.4–2.8%) and fewer upward reclassifications by the alternative equations (1.2–3.1%). Notably, a small number of downward reclassifications were observed with some equations. Although downward reclassifications carry greater clinical relevance than overclassifications, their occurrence was negligible in practical terms: 0.09% for the Martin equation, 0.05% for Sampson, and 0.01% for Rao. Overall, the net gain in correct classification across all alternative equations was modest, ranging from 0.3% to 1.5%. Given such limited improvement, the benefit of replacing the Friedewald equation with these alternatives may not be sufficient to warrant a routine change in clinical practice. A similar conclusion was reached by Drobnki et al. in a recent large-scale study involving 5738 databases, which demonstrated the non-inferiority of the Friedewald equation compared to the widely used Sampson and Martin–Hopkins equations. The study emphasized that, since treatment guidelines are based on trials utilizing Friedewald-estimated LDL-C, and given its comparable performance, replacing it with alternative equations lacks sufficient justification [45].

In the study conducted by Samuel and colleagues, only five equations demonstrated superior overall classification accuracy compared to the Friedewald equation. Notably, their improvement in upward reclassification was significantly more pronounced in samples with triglyceride levels below 400 mg/dL: 19.8% for Martin–Hopkins, 17.6% for Chen (which underperformed in the current study), 17.4% for Delong, 15.4% for Puavilai, and 13.9% for Sampson [11]. As already mentioned, these discrepancies are probably mainly attributable to methodological differences, particularly the choice of reference method: Samuel et al. employed the vertical auto profile ultracentrifugation technique, a more accurate yet less commonly used method in routine clinical practice [11].

### 4.7. Strengths and Limitations

This study presents both strengths and limitations. Among its key strengths is the relatively large sample size: approximately 10,000 lipid profiles were analyzed in Database 1 and over 21,000 in Database 2. While these numbers may not compare with large-scale investigations involving millions of samples, the size of these cohorts is sufficient to detect small effects and provide robust statistical power. Another notable strength lies in the comparison of equations against two different direct LDL-C assays, rather than a single reference, enhancing the generalizability of the findings. Furthermore, the two datasets reflect different clinical settings: Database 1 was derived from a tertiary care hospital, likely representing a predominantly pathological population, whereas Database 2 originated from a private laboratory, capturing a broader spectrum including many individuals undergoing routine testing without known disease.

However, this study is not without limitations. Notably, information on participants’ use of lipid-lowering therapy and body mass index was unavailable (both of which are known to influence lipid levels and could potentially affect the accuracy of LDL-C estimation). Additionally, the reference standard employed was direct LDL-C measurement, rather than β-quantification which is considered the gold standard. Consequently, while comparisons were made to practical clinical assays, the equations were not evaluated against the most analytically accurate method available.

## 5. Conclusions

This study provides a comprehensive, real-world comparison of 24 LDL-C estimation equations in two large, distinct datasets using two direct LDL-C assays as the reference. While widely used equations like Friedewald, Martin–Hopkins, and Sampson showed moderate performance, several lesser-known equations demonstrated better overall accuracy in both bias and categorical classification, especially near the 70 mg/dL clinical threshold. These include the equations by Vujovic, Bauer, Teerakanchana, Orejón, Saiedullah, Dansethakul, and Delong. Nevertheless, all equations occasionally produced extreme estimation errors, highlighting the limitations of relying solely on calculated LDL-C values. Performance varied across datasets and differed from studies using ultracentrifugation, underlining the influence of population characteristics and reference method on equation accuracy.

Although the net improvement over Friedewald was modest, it may be clinically meaningful in risk stratification. However, the smaller gains in one database suggest that switching equations should depend on context. These findings advocate for a tailored, data-driven approach to LDL-C estimation, especially in low LDL-C or high-triglyceride settings where errors are more likely.

## Figures and Tables

**Table 1:** Clinical and laboratory characteristics of the study population.

	Database 1 (*n* = 10,174)			Database 2 (*n* = 21,091)			*p* Value *
	Male	Female	All	Male	Female	All	
Sex (*n*, %)	4958 (48.7%)	5216 (51.3%)	10,174	9995 (47.4%)	11,096 (52.6%)	21,091	0.026
Age (years)	60.0 [49.0–71.0] (0.0–96.0)	62.0 [49.0–72.0] (0.0–97.0)	61.0 [49.0–71.0] (0.0–97.0)	54.0 [43.0–65.0] (1.0–96.0)	58.0 [45.0–67.0] (1.0–94.0)	56.0 [44.0–66.0] (1.0–96.0)	<0.0001
Triglycerides	119.0 [84.8–176.0] (23.6–1745.0)	112.0 [82.2–158.0] (24.6–1943.0)	116.0 [83.0–166.0] (23.6–1943.0)	128.0 [91.0–186.0] (17.0–3490.0)	107.0 [77.0–150.0] (24.0–3690.0)	116.0 [83.0–166.0] (17.0–3690.0)	0.75
Total cholesterol	174.0 [140.0–209.0] (43.8–765.0)	189.0 [156.0–224.0] (59.1–463.0)	182.0 [148.0–217.0] (43.8–765.0)	192.0 [159.0–227.0] (75.0–554.0)	204.0 [172.0–239.0] (33.0–730.0)	198.0 [166.0–233.0] (33.0–730.0)	<0.0001
HDL-cholesterol	44.2 [36.9–53.7] (4.0–189.0)	53.4 [44.4–63.8] (7.1–149.0)	48.9 [39.8–59.6] (4.0–189.0)	44.0 [37.0–51.0] (5.0–123.0)	54.0 [46.0–63.0] (5.0–131.0)	49.0 [41.0–58.0] (5.0–131.0)	0.85
dLDL-cholesterol	107.0 [77.6–138.0] (10.1–388.0)	116.0 [87.2–148.0] (4.3–501.0)	112.0 [82.4–143.0] (4.3–501.0)	125.0 [95.0–158.0] (23.0–472.0)	131.0 [102.0–165.0] (13.0–378.0)	129.0 [99.0–162.0] (13.0–472.0)	<0.0001
Non-HDL-cholesterol	126.7 [93.5–161.6] (24.8–760.9)	131.2 [101.6–167.9] (1.0–403.4)	128.9 [97.9–164.5] (1.0–760.9)	146.0 [114.0–181.0] (39.0–488.0)	147.0 [118.0–182.5] (26.0–696.0)	147.0 [116.0–182.0] (26.0–696.0)	<0.0001
Remnant cholesterol	16.8 [9.9–25.6] (0.0–717.7)	15.2 [9.0–22.9] (0.0–339.5)	15.9 [9.5–24.2] (0.0–717.7)	17.0 [10.0–27.0] (0.0–423.0)	15.0 [8.0–23.0] (0.0–658.0)	16.0 [9.0–24.0] (0.0–658.0)	0.06
TG ≥ 400 mg/dL (*n*, %)	155 (1.52%)	79 (0.77%)	234 (2.29%)	349 (1.65%)	113 (0.53%)	462 (2.18%)	-

Database 1 consists of patients from a tertiary hospital clinical laboratory. Database 2 consists of patients from a private laboratory. All laboratory lipid values are expressed in mg/dL and reported as median [interquartile range], along with the full range of values.). * For database comparison, the following statistical tests were applied: chi-square for sex distribution and paired tests for numerical parameters (paired samples *t*-test and Wilcoxon, depending on statistical distribution). Abbreviations: dLDL—directly measured low-density lipoprotein, HDL—high-density lipoprotein, TG—triglycerides.

**Table 2:** Percentage of patients correctly classified to LDL-C category according to dLDL-C concentration—Database 1.

Equation [Reference]	LDL-C Category (mg/dL)								
	<40 (*n* = 230)	40–54 (*n* = 512)	55–69 (*n* = 847)	70–99 (*n* = 2479)	100–129 (*n* = 2558)	130–159 (*n* = 1986)	160–189 (*n* = 1004)	≥190 (*n* = 558)	Overall (*n* = 10,174)
Ahmadi [21]	33.9	20.5	21.5	37.4	33.0	30.8	29.6	89.1	34.8
Anandaraja [22]	65.7	38.9	41.8	64.1	62.2	60.5	56.5	69.2	59.3
Bauer [23]	65.7	67.6	74.4	86.6	84.0	82.3	82.9	91.0	82.5
Chen [24]	75.2	75.0	69.7	81.1	67.8	54.2	40.9	56.6	65.8
Choi [25]	44.3	42.8	50.5	74.0	76.3	77.9	79.5	97.8	73.0
Cordova [26]	60.4	66.0	57.3	67.6	40.6	21.5	10.8	29.2	43.0
Dansethakul [27]	65.7	67.0	69.7	84.5	82.8	79.8	75.6	79.6	79.4
Delong [28]	79.6	73.6	73.1	84.1	80.5	76.7	72.5	81.5	78.9
Ephraim [29]	87.8	37.1	29.8	57.6	48.6	40.7	32.7	48.6	46.4
Friedewald [7]	88.7	63.9	53.8	74.4	67.2	62.2	54.6	64.7	65.8
Ghasemi [30]	87.8	37.1	29.8	57.6	48.6	40.7	32.7	48.6	46.4
Hattori [31]	91.7	48.4	34.6	60.6	46.5	34.8	23.0	42.8	45.3
Lee & Hu [32]	37.8	40.2	49.6	76.1	75.5	63.4	47.3	55.6	64.6
Martin [15]	81.7	65.4	63.0	81.9	75.6	69.2	59.0	68.6	72.4
Martin extdended [16]	81.7	65.6	63.1	82.0	75.8	69.3	58.6	68.1	72.5
Molavi [33]	88.7	67.4	59.1	75.8	65.8	58.0	48.4	59.7	64.7
Orejón [34]	63.0	67.0	73.3	88.2	85.6	81.5	75.5	81.5	81.7
Puavilai [35]	82.6	73.0	71.3	83.0	79.4	74.6	69.2	78.3	77.4
Rao [36]	82.2	67.0	68.1	81.1	79.8	78.4	77.5	86.4	78.4
Rasouli [37]	66.5	78.1	74.9	78.0	44.9	14.0	2.3	18.6	45.9
Saiedullah [38]	33.5	44.5	64.2	86.8	85.9	81.4	76.7	81.0	79.0
Sampson [14]	87.4	68.6	63.9	80.2	76.1	70.2	62.4	69.9	73.1
Sobhani [39]	90.9	47.7	25.3	51.4	31.4	15.6	5.2	28.9	32.1
Teerakanchana [40]	59.1	60.0	70.5	88.2	87.5	80.0	70.1	75.4	80.4
Vujovic [41]	73.5	72.7	74.7	86.7	83.5	80.9	78.4	86.6	81.9

**Table 3:** Percentage of patients correctly classified to LDL-C category according to dLDL-C concentration—Database 2.

Equation	LDL-C Category (mg/dL)								
	<40 (*n* = 84)	40–54 (*n* = 349)	55–69 (*n* = 966)	70–99 (*n* = 4010)	100–129 (*n* = 5301)	130–159 (*n* = 4837)	160–189 (*n* = 3325)	≥190 (*n* = 2219)	Overall (*n* = 21,091)
Ahmadi [21]	23.8	20.3	18.0	37.9	35.4	32.6	32.6	83.1	38.7
Anandaraja [22]	38.1	29.2	34.2	59.5	59.0	55.9	49.2	61.0	55.3
Bauer [23]	40.5	47.9	58.0	81.3	80.8	77.2	72.4	83.4	77.3
Chen [24]	59.5	69.3	75.1	84.2	69.0	50.3	36.4	47.0	60.4
Choi [25]	26.2	21.2	30.2	64.0	72.2	75.0	74.6	92.8	70.9
Cordova [26]	41.7	62.2	68.7	72.1	37.8	19.9	10.3	23.4	36.2
Dansethakul [27]	34.5	41.0	52.7	79.9	79.6	74.9	68.0	74.1	74.1
Delong [28]	54.8	63.0	65.7	83.8	79.2	71.6	65.0	73.3	74.5
Ephraim [29]	63.1	49.3	45.2	65.5	52.8	41.3	33.1	47.1	48.5
Friedewald [7]	57.1	60.7	59.8	79.3	69.3	58.9	52.0	60.3	64.5
Ghasemi [30]	63.1	49.3	45.2	65.5	52.8	41.3	33.1	47.1	48.5
Hattori [31]	61.9	57.3	50.9	69.5	50.8	34.2	23.5	39.0	45.2
Lee & Hu [32]	22.6	23.8	41.8	70.9	70.3	53.2	36.2	42.1	55.9
Martin [15]	61.9	66.8	70.9	84.2	75.2	64.1	54.0	60.5	69.1
Martin extdended [16]	60.7	67.0	71.0	84.2	75.3	63.7	54.0	60.3	69.0
Molavi [33]	52.4	61.3	61.4	80.0	67.9	54.5	45.1	55.3	61.7
Orejón [34]	35.7	44.1	56.0	82.8	82.1	75.6	67.7	73.6	75.6
Puavilai [35]	57.1	62.8	65.6	83.5	77.8	69.9	62.6	71.4	73.1
Rao [36]	45.2	48.7	51.6	76.8	74.9	72.8	69.1	81.6	73.0
Rasouli [37]	44.0	61.9	70.2	80.9	45.7	15.9	4.5	17.6	37.5
Saiedullah [38]	19.0	21.8	46.3	82.9	84.2	76.6	67.6	74.0	75.5
Sampson [14]	67.9	71.3	67.5	82.5	75.1	65.9	58.3	65.0	70.2
Sobhani [39]	75.0	67.0	45.7	61.3	32.8	15.8	8.2	25.3	31.0
Teerakanchana [40]	41.7	40.1	54.9	81.2	80.6	70.7	59.4	63.8	71.3
Vujovic [41]	52.4	59.3	65.3	83.6	81.1	74.9	68.3	78.1	76.6

## Data Availability

The data presented in this study are available on request from the corresponding author due to privacy reasons.

## Supplementary Materials

The following supporting information can be downloaded at: https://www.mdpi.com/article/10.3390/diagnostics15182298/s1, Table S1. The 24 LDL-C estimation equations; Table S2. Relative performance of 24 LDL-C equations compared with directly measured LDL-C in samples with triglycerides below 400 mg/dL; Table S3. Relative performance of 24 LDL-C equations compared with directly measured LDL-C in samples with triglycerides ≥ 400 mg/dL; Table S4. Classification improvement by the LDL-C equations with superior overall classification accuracy compared with Friedewald (Database 1, *n* = 10,174); Table S5. Classification improvement by the LDL-C equations with superior overall classification accuracy compared with Friedewald (Database 2, *n* = 21,091).

## Abbreviations

The following abbreviations are used in this manuscript:

LDL-C	Low-density lipoprotein cholesterol
FW	Friedwald equation
SN	Sampson equation
MH	Martin–Hopkins equation
TC	Total cholesterol
HDL-C	High-density lipoprotein cholesterol
TG	Triglycerides
dLDL-C	Directly measured low-density lipoprotein cholesterol
NonHDL-C	Non-HDL-cholesterol
RC	Remnant cholesterol
