# Supplementary material for: Comparative Evaluation of 24 LDL-C Estimation Equations Against Direct Assays in Two Independent Cohorts
Source: Diagnostics (Basel). 2025 Sep 10;15(18):2298. doi: 10.3390/diagnostics15182298 (PMC12468580; doi:10.3390/diagnostics15182298)
Supplement: Supplementary file 1 [file diagnostics-15-02298-s001.zip › diagnostics-3776380-supplementary.pdf]

Supplementary Table S1. The 24 LDL-C estimation equations

**Equation**	**LDLC equation**	**Ref.**
Ahmadi	TC / 1.19 + TG / 1.9 − HDLC / 1.1 − 38	[21]
Anandaraja	0.9 × TC − 0.9 × TG / 5 − 28	[22]
Bauer	TC – HDLC – TG / 7.98	[23]
Chen	0.9 × TC – 0.9 × HDLC – 0.1 × TG	[24]
Choi	TC – 0.87 × HDLC – 0.13 × TG	[25]
Cordova	0.7516 × (TC − HDLC)	[26]
Dansethakul	0.9955 × TC – 0.9853 × HDLC – 0.1998 × TG + 7.1449	[27]
Delong	TC − HDLC – 0.16 × TG	[28]
Ephraim	TC – HDLC – TG / 4	[29]
Friedewald	TC − HDLC-C − TG / 5	[7]
Ghasemi	TC – HDLC – TG / 4	[30]
Hattori	0.94 × TC − 0.94 × HDLC − 0.19 × TG	[31]
Lee & Hu	0.75 × TC – 25	[32]
Martin*	TC − HDLC-C − TG / F	[15]
Extended Martin**	TC − HDLC-C − TG / F	[16]
Molavi	0.97 × TC – 0.93 × HDLC – 0.19 × TG	[33]
Orejón	0.974 × TC – 0.968 × HDLC – 0.160 × TG + 5.361	[34]
Puavilai	TC − HDLC − TG / 6	[35]
Rao	(4.7 × TC – 4.364 × HDLC – TG) / 4.487	[36]
Rasouli	0.75 × TC -0.5 × HDLC – 0.1 × TG	[37]
Saiedullah	TC – HDLC – TG / 5 + 15.3 × TG / TC – 2.4	[38]
Sampson	TC / 0.948 − HDLC / 0.971 − (TG / 8.56 + TG × non-HDLC / 2140 − TG^2 / 16100) − 9.44	[14]
Sobhani	LDL-C = TC − HDLC − TG / 6.7	[39]
Teerakanchana	LDL-C = 0.91 × TC – 0.634 × HDLC – 0.111 × TG – 6.755	[40]
Vujovic	LDL-C = TC − HDLC − TG / 6.85	[41]

* The Martin/ Hopkins equation uses an adjustable factor F based on triglyceride and non-HDLC levels.

** The extended Martin/ Hopkins equation increases the triglyceride (TG) range for LDL-C estimation from a maximum of 400 mg/dL in the original equation to up to 800 mg/dL.

Supplementary Table S2. Relative performance of 24 LDL-C equations compared with directly measured LDL-C in samples with triglycerides below 400 mg/dL.

	Database 1 (n=9,940)						Database 2 (n=20,629)					
	**Median**	**IQR**	**Range**	**Δ% Median**	**Δ% IQR**	**Δ% Range**	**Median**	**IQR**	**Range**	**Δ% Median**	**Δ% IQR**	**Δ% Range**
dLDL-C (ref.)	112.0	82.4-143.0	4.3-501.0	-	-	-	129.0	99.0-162.0	13.0-472.0	-	-	-
Ahmadi	129.0	91.1-174.1	-22.3 to 446.1	12.4	-6.4 to 40.3	-115.3 to 952.9	144.3	107.2-189.6	1.6-562.1	8.1	-7.8 to 32.4	-93.8 to 1167.0
Anandaraja	111.6	83.4-141.2	-1.5 to 364.0	-1.7	-9.6 to 7.7	-107.8 to 315.9	126.6	99.2-156.1	-22.2 to 451.8	-3.4	-10.8 to 6.1	-271.0 to 217.1
Bauer	112.2	83.1-144.4	-4.5 to 384.9	0.5	-2.7 to 4.2	-112.6 to 527.5	129.9	101.3-161.6	7.7-474.9	0.4	-3.4 to 5.5	-76.0 to 226.0
Chen	102.3	76.3-131.7	-3.5-349.8	-8.1	-11.1 to -4.4	-102.4 to 478.5	118.4	92.7-147.4	11.0-428.8	-8.3	-11.9 to -3.5	-76.5 to 195.5
Choi	118.3	89.4-150.5	5.3-392.8	6.1	2.9-10.2	-92.1 to 527.8	135.9	107.4-167.8	9.1-483.0	5.2	0.7-11.1	-71.4 to 231.2
Cordova	96.1	73.1-122.2	0.7-314.0	-14.3	-18.6 to -7.9	-99.4 to 473.4	109.7	87.1-135.2	19.5-366.7	-15.5	-19.9 to -9.09	-68.3 to 171.7
Dansethakul	109.8	81.9-140.7	-1.8 to 371.4	-0.8	-4.5 to 2.8	-107.0 to 473.0	127.5	99.6-158.4	-8.3 to 472.8	-1.0	-5.4 to 4.1	-164.4 to 220.4
Delong	107.7	79.1-139.5	-6.1 to 375.8	-3.1	-6.5 to 0.2	-185.8 to 490.0	125.4	97.3-156.8	-3.0 to 471.3	-2.9	-6.6 to 1.8	-123.6 to 220.3
Ephraim	96.2	69.0-127.0	-20.7 to 354.6	-12.5	-18.3 to -8.2	-375.7 to 392.6	114.2	86.5-145.0	-31.2 to 462	-11.0	-16.1 to -6.1	-340.3 to 205.4
Friedewald	102.8	74.6-133.7	-9.8 to 365.4	-7.4	-11.5 to -3.8	-270.2 to 446.7	120.4	92.6-151.6	-15.6 to 467.2	-6.6	-10.7 to -2.0	-220.0 to 213.7
Ghasemi	96.2	69.0-127.0	-20.7 to 354.6	-12.5	-18.3 to -8.2	-375.7 to 392.6	114.2	86.5-145.0	-31.2 to 462	-11.0	-16.1 to -6.1	-340.3 to 205.4
Hattori	96.4	70.0-125.4	-9.6 to 342.9	-13.1	-17.0 to -9.8	-264.2 to 411.7	113.0	86.7-142.1	-15.2 to 438.9	-12.4	-16.3 to -8.0	-217.6 to 194.5
Lee & Hu	110.7	85.2-136.2	7.8-341.0	-2.3	-8.4 to 6.6	-58.9 to 402.0	122.7	99.5-149.0	-0.2 to 390.5	-5.5	-11.9 to 3.33	-101.1 to 187.0
Martin *	105.2	77.5-136.0	-11.7 to 371.0	-5.8	-9.2 to -2.3	-254.0 to 496.6	122.2	94.8-152.7	14.0-464.8	-5.6	-9.2 to -0.9	-80.1 to 212.3
Molavi	102.2	74.9-132.3	-5.1 to 358.2	-7.8	-11.5 to -4.4	-212.2 to 436.3	119.4	92.2-149.6	-13.0 to 456.2	-7.4	-11.5 to -2.7	-200.0 to 206.7
Orejón	110.1	82.2-140.9	-0.0 to 370.8	-0.9	-4.1 to 2.5	-100.0 to 492.2	127.2	99.9-157.8	1.1-464.4	-1.2	-5.2 to 3.8	-90.8 to 217.3
Puavilai	106.9	78.3-138.5	-6.4 to 374.1	-3.8	-7.3 to -0.4	-199.9 to 482.7	124.5	96.5-155.8	-5.1 to 470.6	-3.5	-7.3 to 1.1	-139.7 to 219.2
Rao	109.8	80.4-142.1	-6.6 to 384.5	-0.7	-5.1 to 3.07	-223.2 to 461.3	128.4	99.0-160.9	-18.8 to 492.9	-0.4	-5.1 to 4.8	-245.2 to 229.8

Rasouli	96.4	74.3-120.7	11.1-304.7	-13.6	-17.0 to -8.9	-81.1 to 374.5	109.5	87.8-133.7	9.45-372.1	-15.2	-19.5 to -9.4	-72.8 to 154.9
Saiedullah	110.9	84.2-141.4	-4.9 to 371.2	-0.2	-3.6 to 4.5	-103.3 to 520.5	127.8	100.9-158.1	30.7-467.6	-0.7	-4.7 to 4.7	-62.7 to 295.4
Sampson	105.4	76.9-136.7	-10.3 to 360.4	-5.5	-9.1 to -2.08	-267.9 to 464.2	123.0	95.0-154.1	3.3-471.7	-4.9	-8.7 to -0.3	-82.3 to 214.2
Sobhani	92.0	67.4-119.5	-11.5 to 323.8	-17.3	-20.4 to -14.0	-191.4 to 427.1	107.3	83.1-134.5	3.0-402.4	-17.0	-20.1 to -13.0	-82.8 to 175.5
Teerakanchana	110.2	83.4-139.6	6.4-363.7	-1.5	-4.66 to 2.7	-83.7 to 458.4	126.0	99.6-155.4	7.3-444.0	-2.6	-7.1 to 3.3	-73.2 to 202.4
Vujovic	109.5	80.8-141.5	-5.5 to 379.5	-1.7	-4.9 to 1.7	-156.2 to 505.1	127.2	98.9-158.8	1.3-472.8	-1.5	-5.3 to 3.3	-89.9 to 222.6

* The extended Martin equation was not included in this table because it is applicable only when triglyceride (TG) levels are ≥400 mg/dL, whereas the data presented here include only entries with TG <400 mg/dL.

Supplementary Table S3. Relative performance of 24 LDL-C equations compared with directly measured LDL-C in samples with triglycerides ≥ 400 mg/dL.

	Database 1 (n=234)						Database 2 (n=462)					
	**Median**	**IQR**	**Range**	***Δ% Median***	***Δ% IQR***	***Δ% Range***	**Median**	**IQR**	**Range**	***Δ% Median***	***Δ% IQR***	***Δ% Range***
dLDL-C (ref.)	121.0	83.0-154.0	10.1-295.0	-	-	-	112.5	83.0-432.0	23.0-376.0	-	-	-
Ahmadi	425.1	360.6-519.9	254.3-1519.5	246.7	165.5-431.8	62.7-5485.1	420.9	367.3-524.0	268.3-2249.4	270.4	181.0-467.8	52.0-8692.1
Anandaraja	84.8	47.6-130.2	-102.5 to 346.4	-28.6	-48.4 to -14.7	-644.5 to 701.8	87.3	49.9-123.3	-298.0 to 321.7	-24.2	-43.0 to -11.3	-884.2 to 167.7
Bauer	136.1	99.3-176.5	-41.6 to 542.2	8.0	-1.9 to 23.4	-274.7 to 1155.2	133.8	103.3-169.7	-61.0 to 401.8	13.2	1.6 to 30.4	-230.0 to 748.2
Chen	130.4	96.9-166.4	-27.2 to 510.3	3.4	-5.9 to 21.4	-214.4 to 1081.3	128.1	98.7-158.8	-24.0 to 372.9	7.7	-2.6 to 26.5	-143.0 to 763.6
Choi	136.8	99.5-177.5	-42.9 to 534.6	9.4	-0.7 to 22.9	-280.4 to 1137.5	135.9	101.4-172.6	-69.0 to 402.1	14.4	3.3-31.8	-266.9 to 723.0
Cordova	160.2	134.7-188.8	43.8-571.9	25.9	10.6-61.5	-14.3 to 1223.8	160.0	133.7-188.6	47.3-523.1	34.7	13.5-75.5	-16.2 to 1641.7
Dansethakul	97.5	57.8-142.4	-93.9 to 416.0	-19.4	-35.6 to -6.1	-494.8 to 863.0	99.9	61.0-134.3	-318.7 to 368.0	-14.8	-32.4 to -2.0	-938.7 to 199.6
Delong	114.7	74.2-157.0	-69.2 to 481.7	-8.0	-18.0 to 5.7	-391.0 to 1015.1	114.5	78.9-148.7	-177.4 to 380.2	-2.0	-17.6 to 11.0	-566.8 to 476.0
Ephraim	65.9	16.5-108.6	-141.1 to 328.2	-44.3	-78.1 to -27.8	-857.9 to 651.5	65.1	23.0-102.5	-509.5 to 340.5	-43.0	-72.2 to -26.8	-1440.7 to 156.3
Friedewald	90.6	51.3-136.0	-101.2 to 411.9	-24.8	-43.0 to -10.4	-525.2 to 853.5	93.3	54.2-127.8	-325.0 to 362.6	-20.6	-38.0 to -6.8	-955.2 to 194.9
Ghasemi	65.9	16.5-108.6	-141.1 to 328.2	-44.3	-78.1 to -27.8	-857.9 to 651.5	65.1	23.0-102.5	-509.5 to 340.5	-43.0	-72.2 to -26.8	-1440.7 to 156.3
Hattori	84.2	46.8-126.4	-96.7 to 383.7	-30.3	-47.5 to -16.5	-506.4 to 788.2	86.4	49.8-119.1	-312.8 to 339.6	-26.6	-42.9 to -13.2	-923.3 to 175.6
Lee & Hu	159.1	134.7-191.0	32.6-548.7	26.9	12.3-60.4	-13.18 to 1170.2	161.7	135.5-192.5	62.7-522.5	36.1	16.7-76.4	-16.1 to 1620.6
Martin	129.5	94.3-162.4	-8.6 to 500.4	6.9	-3.9 to 19.0	-136.3 to 1058.5	129.3	98.7-157.7	-137.7 to 385.0	8.3	-1.7 to 26.0	-462.4 to 560.3

Martin extended*	125.5	94.3-158.1	7.2-500.5	3.6	-6.3 to 17.2	-69.5 to 1058.5	125.5	97.9-151.7	-137.7 to 380.8	6.8	-4.7 to 23.4	-462.4 to 560.3
Molavi	91.6	55.4-136.0	-94.2 to 406.7	-24.3	-40.9 to -10.5	-495.9 to 841.4	93.7	55.8-127.1	-299.4 to 354.5	-19.3	-36.9 to -7.0	-888.1 to 190.3
Orejón	114.0	74.9-156.0	-65.3 to 467.3	-8.0	-16.7 to 5.1	-374.4 to 981.7	114.5	79.8-147.2	-182.6 to 374.0	-2.0	-16.4 to 10.9	-580.6 to 443.0
Puavilai	110.1	69.4-153.2	-74.6 to 470.1	-10.4	-21.4 to 2.6	-413.4 to 988.1	110.8	74.8-144.3	-202.0 to 377.3	-5.7	-20.5 to 7.2	-631.5 to 423.6
Rao	91.3	45.2-137.8	-115.3 to 408.4	-24.8	-46.6 to -8.5	-606.3 to 845.5	92.0	51.5-129.4	-387.9 to 375.8	-21.0	-41.5 to -6.0	-1120.7 to 200.8
Rasouli	106.6	77.2-137.8	-31.4 to 397.2	-14.7	-22.6 to -4.1	-213.9 to 819.4	105.8	79.1-133.9	-53.6 to 304.6	-10.6	-18.8 to 2.6	-239.4 to 511.3
Saiedullah	124.8	92.2-161.8	32.8-444.4	4.5	-5.3 to 17.8	-49.3 to 928.7	124.9	95.6-154.4	-198.5 to 374.3	6.5	-3.9 to 21.9	-622.3 to 320.2
Sampson	106.2	74.5-141.0	-22.8 to 317.0	-12.3	-20.3 to -2.0	-195.9 to 420.4	106.6	78.3-135.3	-40.3 to 334.3	-7.9	-18.3 to 4.2	-206.0 to 240.3
Sobhani	109.6	76.7-143.1	-42.7 to 451.2	-12.1	-21.3 to -0.4	-279.7 to 944.4	108.2	80.6-138.6	-73.1 to 335.7	-8.4	-18.5 to 5.6	-289.1 to 562.0
Teerakanchana	127.4	94.5-164.8	-37.1 to 493.1	1.5	-7.2 to 15.6	-255.9 to 1041.4	126.6	96.9-159.8	-46.6 to 370.9	6.9	-3.2 to 22.9	-188.4 to 702.6
Vujovic	122.0	84.5-164.0	-58.1 to 506.1	-1.0	-11.3 to 12.3	-344.1 to 1071.7	122.6	87.5-156.7	-125.6 to 386.4	3.2	-9.5 to 18.7	-430.7 to 585.9

* For the extended Martin equation, factors were derived from the 280-cell table version [16]. For database entries in which the extended equation was not applicable (TG ≥800 mg/dL), the factor from the original Martin equation [15] was used.

Supplementary Table S4. Classification improvement by the LDL-C equations with superior overall classification accuracy compared with Friedewald (Database 1, n=10174)

	**Better classification by Friedewald**	**Same class**	**Upward reclassification**	**Downward reclassification**	**Net gain in correct classification**
Bauer	146 (1.44%)	9509 (93.46%)	519 (5.10%)	0 (0.00%)	373 (3.66%)
Choi	410 (4.03%)	9158 (90.01%)	606 (5.96%)	0 (0.00%)	196 (1.93%)
Dansethakul	133 (1.31%)	9634 (94.69%)	407 (4.00%)	0 (0.00%)	274 (2.69%)
Delong	45 (0.44%)	9816 (96.48%)	313 (3.08%)	0 (0.00%)	268 (2.64%)
Martin	98 (0.96%)	9788 (96.21%)	288 (2.83%)	0 (0.00%)	190 (1.87%)
Martin extended	97 (0.95%)	9789 (96.21%)	288 (2.83%)	0 (0.00%)	191 (1.87%)
Orejón	137 (1.35%)	9557 (93.93%)	480 (4.72%)	0 (0.00%)	343 (3.37%)
Puavilai	33 (0.32%)	9871 (97.03%)	270 (2.65%)	0 (0.00%)	237 (2.33%)
Rao	66 (0.65%)	9791 (96.23%)	317 (3.12%)	0 (0.00%)	251 (2.47%)
Saiedullah	281 (2.76%)	9356 (91.96%)	537 (5.28%)	0 (0.00%)	256 (2.52%)
Sampson	33 (0.32%)	9951 (97.81%)	190 (1.87%)	0 (0.00%)	157 (1.55%)
Teerakanchana	203 (2.00%)	9440 (92.78%)	531 (5.22%)	0 (0.00%)	328 (3.22%)
Vujovic	83 (0.82%)	9662 (94.96%)	429 (4.22%)	0 (0.00%)	346 (3.40%)

Only those LDL-C equations that demonstrated superior overall classification accuracy (as presented in Table 2) were selected for comparison with the Friedewald equation. Classification performance was evaluated specifically at the 70 mg/dL threshold. The net gain in correct classification was defined as the number of instances where the alternative equation correctly reclassified LDL-C values that were misclassified by the Friedewald equation (i.e., both upward and downward reclassifications), minus the number of cases where the Friedewald equation had correctly classified LDL-C values and the alternative equation did not.

Supplementary Table S5. Classification improvement by the LDL-C equations with superior overall classification accuracy compared with Friedewald (Database 2, n=21091)

	**Better classification by Friedewald**	**Same class**	**Upward reclassification**	**Downward reclassification**	**Net gain in correct classification**
Bauer	290 (1.37%)	20198 (95.77%)	603 (2.86%)	0 (0.00%)	313 (1.49%)
Choi	599 (2.84%)	19833 (94.04%)	659 (3.12%)	0 (0.00%)	60 (0.28%)
Dansethakul	257 (1.22%)	20400 (96.72%)	434 (2.06%)	0 (0.00%)	177 (0.84%)
Delong	117 (0.55%)	20574 (97.55%)	400 (1.90%)	0 (0.00%)	283 (1.35%)
Martin	137 (0.65%)	20549 (97.43%)	386 (1.83%)	19 (0.09%)	268 (1.27%)
Martin extended	138 (0.65%)	20549 (97.43%)	385 (1.83%)	19 (0.09%)	266 (1.26%)
Orejón	267 (1.27%)	20277 (96.14)	547 (2.59%)	0 (0.00%)	280 (1.32%)
Puavilai	93 (0.44%)	20651 (97.91%)	347 (1.65%)	0 (0.00%)	254 (1.21%)
Rao	204 (0.97%)	20577 (97.56%)	308 (1.46%)	2 (0.01%)	106 (0.50%)
Saiedullah	430 (2.04%)	20018 (94.91%)	643 (3.05%)	0 (0.00%)	213 (1.01%)
Sampson	70 (0.33%)	20755 (98.41%)	256 (1.21%)	10 (0.05%)	196 (0.93%)
Teerakanchana	331 (1.57%)	20189 (95.72%)	571 (2.71%)	0 (0.00%)	240 (1.14%)
Vujovic	164 (0.78%)	20431 (96.87%)	496 (2.35%)	0 (0.00%)	332 (1.57%)

Only those LDL-C equations that demonstrated superior overall classification accuracy (as presented in Table 3) were selected for comparison with the Friedewald equation. Classification performance was evaluated specifically at the 70 mg/dL threshold. The net gain in correct classification was defined as the number of instances where the alternative equation correctly reclassified LDL-C values that were misclassified by the Friedewald equation (i.e., both upward and downward reclassifications), minus the number of cases where the Friedewald equation had correctly classified LDL-C values and the alternative equation did not.
